# The Future of Freshwater Macrophytes in a Changing World: Dissolved Organic Carbon Quantity and Quality and Its Interactions With Macrophytes

**DOI:** 10.3389/fpls.2018.00629

**Published:** 2018-05-14

**Authors:** Rosanne E. Reitsema, Patrick Meire, Jonas Schoelynck

**Affiliations:** Ecosystem Management Research Group (Ecobe), Department of Biology, University of Antwerp, Antwerp, Belgium

**Keywords:** aquatic plants, DOC, climate change, humic substances, freshwater ecology, CO_2_

## Abstract

Freshwater ecosystems are confronted with the effects of climate change. One of the major changes is an increased concentration of aquatic carbon. Macrophytes are important in the aquatic carbon cycle and play as primary producers a crucial role in carbon storage in aquatic systems. However, macrophytes are affected by increasing carbon concentrations. The focus of this review lies on dissolved organic carbon (DOC), one of the most abundant forms of carbon in aquatic ecosystems which has many effects on macrophytes. DOC concentrations are rising; the exact cause of this increase is not known, although it is hypothesized that climate change is one of the drivers. The quality of DOC is also changing; for example, in urban areas DOC composition is different from the composition in natural watersheds, resulting in DOC that is more resistant to photo-degradation. Plants can benefit from DOC as it attenuates UV-B radiation, it binds potentially harmful heavy metals and provides CO_2_ as it breaks down. Yet plant growth can also be impaired under high DOC concentrations, especially by humic substances (HS). HS turn the water brown and attenuate light, which limits macrophyte photosynthesis at greater depths. This leads to lower macrophyte abundance and lower species diversity. HS form a wide class of chemicals with many different functional groups and they therefore have the ability to interfere with many biochemical processes that occur in freshwater organisms. Few studies have looked into the direct effects of HS on macrophytes, but there is evidence that HS can interfere with photosynthesis by entering macrophyte cells and causing damage. DOC can also affect reactivity of heavy metals, water and sediment chemistry. This indirectly affects macrophytes too, so they are exposed to multiple stressors that may have contradictive effects. Finally, macrophytes can affect DOC quality and quantity as they produce DOC themselves and provide a substrate to heterotrophic bacteria that degrade DOC. Because macrophytes take a key position in the aquatic ecosystem, it is essential to understand to what extent DOC quantity and quality in surface water are changing and how this will affect macrophyte growth and species diversity in the future.

## Introduction

Like many ecosystems, freshwater ecosystems are confronted with the effects of climate change (Hossain et al., 2016). One of the major changes is an increased concentration of C in the water (Evans et al., 2005; Hasler et al., 2016; Williams et al., 2016). Research in this regard mostly focusses on ocean acidification: decreasing ocean pH caused by uptake of atmospheric CO_2_, which is currently rising because of emission by human activities (Doney et al., 2009). The consequences for fauna and flora are well studied: e.g., coral diversity decreases at a lower pH, whereas non-calcareous algae benefit (Fabricius et al., 2011). Less research, however, has been done in freshwater ecosystems and consequences are less well understood. A recently published review paper concluded that the effects of elevated atmospheric CO_2_ levels on freshwater CO_2_ levels have not been clearly demonstrated (Hasler et al., 2016). ‘Freshwater acidification’ due to climate change is likely not comparable to acidification in oceans since CO_2_ concentrations in most freshwater ecosystems are currently already several times higher than in the oceans, but water bodies with a relatively low CO_2_ concentration can be expected to acidify. Moreover, degradation of DOC (dissolved organic carbon) has been mentioned as a potential alternative driver of CO_2_ concentrations in freshwater (Sobek et al., 2003). In addition, DOC can affect aquatic ecosystems in various ways; for example by attenuating light (Karlsson et al., 2009) and by interfering with biochemical processes within aquatic organisms (Steinberg et al., 2008). Although DOC is not always taken into account when determining aquatic system characteristics (such as trophic status), DOC concentrations can provide information about how aquatic systems may react to contaminants and global warming (Williamson et al., 1999). An increased DOC concentration can have multiple effects on macrophyte productivity (Steinberg et al., 2006) and hence on the entire food web and ecosystem.

The goal of this review is to: (i) give an overview of CO_2_ and DOC concentrations and origins in freshwater ecosystems and summarize possible explanations for the rise in DOC concentrations that is being observed in many waterbodies, (ii) summarize the direct and indirect effects of DOC on macrophytes, (iii) explain how macrophytes affect aquatic carbon themselves, (iv) discuss how C cycling and macrophytes are affected by the interaction between changing DOC and other effects of climate change, and (v) identify research gaps with regard to those four topics.

## CO_2_ and DOC in Freshwater Ecosystems

There are different forms and interactions of aquatic C (see **Box 1**). Two of those forms, CO_2_ and DOC, have the most direct interaction with macrophytes and are therefore discussed in more detail.

**BOX 1:**
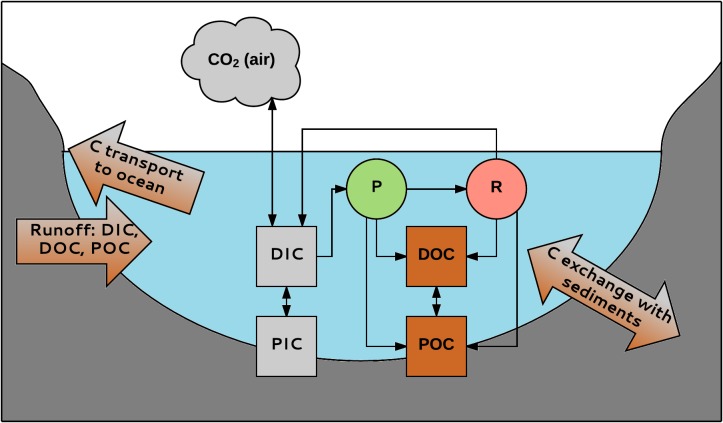
Different forms and interactions of aquatic carbon. Inland waters primarily receive C from terrestrial ecosystems (Thomas, 1997). This C (1.9 Pg C y^-1^) is transported to the oceans (0.9 Pg C y^-1^), buried in the sediments (0.2 Pg C y^-1^) or emitted as CO_2_ (0.8 Pg C y^-1^) (Cole et al., 2007). More recent estimations are different: Raymond et al. (2013) claims that CO_2_ emission from inland waters can be as high as 2.1 Pg C y^-1^. Aquatic C occurs in different forms. Firstly, a division is made between organic and inorganic C. Organic C is a mixture of organic compounds originating from detritus or primary producers. It can be divided into POC (particulate organic carbon; particles > 0.45 μm) and DOC (dissolved organic carbon; particles < 0.45 μm). DOC usually makes up 90% of the total amount of aquatic organic C. Its concentration ranges from 0.1 to >300 mg L^-1^ (Sobek et al., 2007). Likewise, inorganic C also consists of a particulate (PIC) and a dissolved phase (DIC). PIC mainly consists of carbonates (e.g., CaCO_3_), DIC consists of carbonate (CO_3_^2-^), bicarbonate (HCO_3_^-^), CO_2_ and a negligibly small fraction of carbonic acid (H_2_CO_3_). The inorganic C compounds exist in equilibrium that depends on the pH of the water (Stumm and Morgan, 1996). DIC concentrations in freshwater range from about zero in acidic waters to 60 mg C L^-1^ in areas with carbonate-rich sediments (Madsen and Sand-Jensen, 1991). POC can be degraded to form DOC; DOC can become POC by flocculation. Inorganic and organic C are linked through aquatic organisms. CO_2_ is used in photosynthesis (P) by for instance macrophytes, produced by respiration (R), and exchanged with the atmosphere. Organic C is produced by organisms and is released during and after their life; e.g., in rivers, 1–20% of the total amount of DOC is produced by macrophytes (Thomas, 1997). Carbon can enter the system from the catchment and is transported to the oceans by rivers and streams. There is also exchange with C in the sediments, e.g., burial of organic carbon, which is important for C sequestration in aquatic habitats (Regnier et al., 2013). Aquatic systems are very important in global C sequestration; e.g., when different European ecosystems are compared, inland aquatic systems form the second largest C sink (19–41 Tg C y^-1^); only forests take up more C (125–223 Tg C y^-1^) (Luyssaert et al., 2012).

### CO_2_

In 2017 the average atmospheric CO_2_ concentration was 406 ppm (Tans and Keeling, 2018) and it has been predicted that this value may increase to over 1000 ppm by the year of 2100 (IPCC, 2013). However, the concentration of CO_2_ in the atmosphere is lower than in most freshwater systems, which are supersaturated with CO_2_ and act as CO_2_ sources to the atmosphere (contrary to oceans which are sinks). Raymond et al. (2013) found that in 95% of the over 6500 stream and river sampling points they studied, the median CO_2_ partial pressure (*p*CO_2_) was larger than atmospheric CO_2_ levels. The average of the medians in rivers and streams was 3100 ppm and in freshwater lakes it was 1120 ppm.

Increasing atmospheric CO_2_ concentrations will only have a small effect on the concentration of CO_2_ in the water and will likely not lead to acidification on the scale observed in oceans. Phillips et al. (2015) calculated hypothetical pH decrease in freshwater lakes with different CO_2_ concentrations under rising atmospheric CO_2_ concentrations. If the CO_2_ concentration in the air rises to 800 ppm, in an average lake with a CO_2_ concentration of 1100 ppm, the pH will decrease by 0.14. The calculated changes in pH caused by increased CO_2_ normally depend on the alkalinity; systems with low alkalinity may be more vulnerable to acidification caused by increased CO_2_ concentrations and systems with a high alkalinity may be less vulnerable (Stets et al., 2017). However, the change in pH in the calculation by Phillips et al. (2015) was independent of alkalinity if this fell between 800 and 2500 meq m^-3^, although the initial pH of the water was determined by alkalinity. The study by Phillips et al. (2015) focussed on the Laurentian Great Lakes, but in other freshwater systems the effect on pH may be different. Alkalinity may play a more prominent role and other factors can affect the pH, such as the sediment, photosynthesis, and respiration in the water, water influx and land use. Since those factors can be highly variable both in time and space, the effect of increased CO_2_ on pH is more difficult to predict than in oceans (Hasler et al., 2017). IPCC (2007) predicted that the global average decrease of the pH in oceans will be 0.35 if the concentration of CO_2_ in the air rises to 800 ppm, a larger value than predicted for the Laurentian Great Lakes. For rivers, possible decreases in pH as a result of rising atmospheric CO_2_ concentrations have not been calculated, but it can be expected that this will be even lower than in lakes, as rivers have on average a higher CO_2_ concentration.

There can be substantial variation in the amount of CO_2_ in freshwater systems, depending on, for example, characteristics of the catchment soil (Manahan, 2000), discharge from the catchment (McDonald et al., 2013) and the season and time of the day. Seasonal variation is caused by high autotrophic productivity in summer and autumn compared to winter and spring (Dawson et al., 2009). Autotrophic organisms can also cause daily fluctuations in the concentration of CO_2_. In productive lakes, the concentration of CO_2_ can decrease to near zero during the day and is restored during the night, when no photosynthesis takes place (Maberly, 1996). Another important driver of aquatic CO_2_ concentrations is degradation of DOC. DOC is converted to CO_2_ by photo-degradation caused by UV light and to a smaller extent by microbial respiration (Goulsbra et al., 2016). The rate of DOC degradation highly depends on the type DOC: chromophoric structures in DOC are degraded most easily by UV light (Jones et al., 2015), even though microbes can degrade colored DOC as well and mainly respire it instead of incorporating it into their biomass. Protein-like DOC is most readily degraded by microbes (Berggren and del Giorgio, 2015). However, the rate of microbial degradation depends on nutrient availability (Jones et al., 2015). Raymond et al. (2013) estimated that global inland freshwater ecosystem CO_2_ emissions amount to 2.1 Pg C per year. In comparison, anthropogenic total emission of CO_2_ in the year 2000 was 8.03 Pg C per year (IPCC, 2013).

During photosynthesis, macrophytes take up inorganic C, primarily CO_2_. Even though freshwater systems are usually supersaturated with CO_2_ (Raymond et al., 2013), photosynthesis may still be limited since (i) diffusion of CO_2_ in water occurs 10^4^ times more slowly than in air (Maberly and Spence, 1989), and (ii) in highly productive environments with slow water flow velocity, the pH of the water is raised by photosynthesis, which reduces availability of CO_2_ (Maberly and Spence, 1983). In order to maintain net photosynthesis macrophytes have evolved four different strategies. First, there are submerged macrophyte species that can develop aerial leaves that can take up atmospheric CO_2_ (Maberly and Spence, 1989). Secondly, some species can take up CO_2_ from the sediments if they have a suitable morphology, i.e., sufficient root development and high tissue porosity (Winkel and Borum, 2009). The third strategy is utilizing HCO_3_^-^ instead of CO_2_ as an inorganic C source; a strategy used by 50% of all macrophytes (Madsen and Sand-Jensen, 1991). The fourth strategy to prevent photosynthesis limitation because of C deficit is using an alternative form of photosynthesis than the common C_3_ pathway. There are macrophyte species with C_4_ or CAM metabolism, although this is not widespread and can occur simultaneously with HCO_3_^-^ use. Since both HCO_3_^-^ and C_4_/CAM metabolism are a costly process, their use is often phenotypically plastic (Madsen and Sand-Jensen, 1991).

### Dissolved Organic Carbon (DOC)

The terms DOC (dissolved organic carbon) and DOM (dissolved organic matter) are often used interchangeably, but in fact, DOC is a quantification of DOM; approximately 67% of DOM consists of C (Bolan et al., 2011). In this review, the term DOC will be used. DOC consists of a diverse mixture of compounds with a molecular weight from 100 to 100,000 daltons. The compounds have wide variety of chemical functional groups like amide, carboxyl, hydroxyl and ketone groups (Leenheer and Croué, 2003). The main part of DOC (60–90%) consists of humic substances (HS) (Sachse et al., 2005). HS consist of plant- or animal material from which readily bioconsumable parts have been removed (Frimmel, 2005). HS are relatively complex molecules that do not have a standard chemical formula, in contrast to non-humic substances (such as carbohydrates, lipids, and amino acids). There is a subdivision of HS into fulvic acid, humic acid and humin (Pettit, 2004). This subdivision is based on solubility in water with different degrees of acidity. HS have a relatively high molecular weight and they have a yellow to black color often causing brownification of the water (Findlay and Sinsabaugh, 2003).

Other major classes of DOC are hydrophilic acids (high molecular weight) and compounds with a low molecular weight: carbohydrates, carboxylic acids, and amino acids (Findlay and Sinsabaugh, 2003). Although these substances can serve as food- (carbohydrates, amino acids) or information source [amino acids and carboxylic acids (Thomas, 1997)] to aquatic organisms, there are no known effects on freshwater macrophytes, so the main focus of this review is on HS.

Dissolved organic carbon in freshwater systems can originate from allochthonous and autochthonous sources, but usually there is a larger contribution of allochthonous DOC (Thomas, 1997). Allochthonous DOC mainly comes from terrestrial plant material (Steinberg et al., 2006) and enters rivers and lakes after precipitation has flowed through vegetation and/or the soil (Findlay and Sinsabaugh, 2003). Autochthonous DOC is produced by algae (usually phytoplankton in lentic systems and periphyton in lotic systems) and macrophytes (Findlay and Sinsabaugh, 2003) and is in general more labile than allochthonous DOC (Williamson et al., 1999). DOC concentrations can vary on different scales. On a large scale, DOC concentrations tend to be higher with more peatland area in the catchment, more precipitation and if water that enters a river or stream has flowed through organic-rich soil (Findlay and Sinsabaugh, 2003). There are also differences on a smaller scale. DOC concentrations are usually highest in the pore water and lowest in the water column. At the air-water interface, intermediate concentrations are found; however, HS are degraded by UV-radiation, so its share in the DOC concentration is lower at the air-water interface. The higher concentrations of DOC in the pore water and at the air-water interface can be explained by the higher densities of detritivores and increased exposal to UV radiation, respectively, compared to the water column (Thomas, 1997).

### Increasing DOC Concentrations and Changing DOC Quality

**FIGURE 1:**
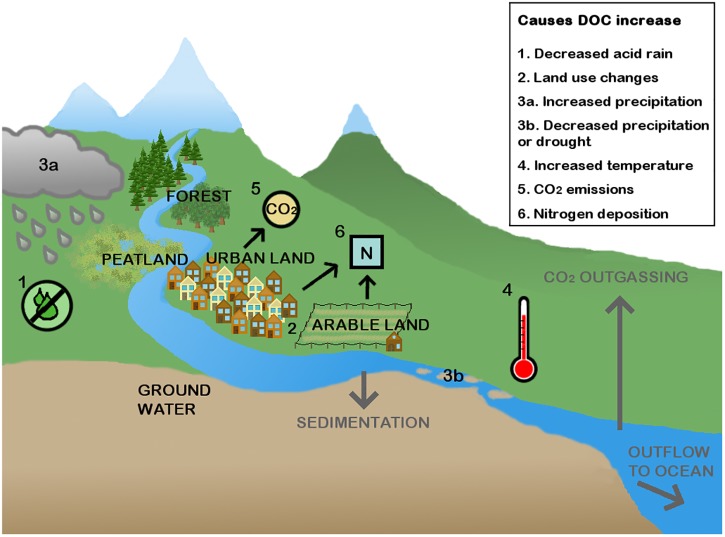
The DOC sources in a river. DOC enters the rivers mainly from the terrestrial system, especially peatlands can release substantial amounts of DOC, but it can also come from other natural systems such as forests and through ground water seepage. Additionally, DOC also comes from urban and arable land. Six main causes of rises in DOC are shown. DOC leaves the system when it is incorporated in the sediments, degraded into CO_2_, or transported to the oceans.

Since the 1990s an increase in DOC has been observed in European and North American rivers and lakes; between 1990 and 2004 concentrations increased by up to 0.15 mg L^-1^ y^-1^ (Monteith et al., 2007). The increases have been observed in acid sensitive rivers and lakes and appear to be present in both waters that already had a relatively high DOC concentration and waters that initially had a low DOC concentration (Evans et al., 2005). Data on long-term DOC trends in other parts of the world is scarce, but increasing DOC concentrations have also been reported, for example in Lake Jaisamand in India (Pandey and Pandey, 2012) and it has been suggested that DOC concentrations have increased in Lake Paldang in South Korea (Kang et al., 2010). The exact cause of this rise in DOC has not been found, though it has been suggested that an interaction between several mechanisms is responsible (Sucker and Krause, 2010). In **Figure 1** a graphical overview of current explanations for increased allochthonous DOC is shown. The main cause appears to be decreased atmospheric deposition of sulfur (acid rain) (Pagano et al., 2014). Anthropogenic SO_2_ emissions led to acidification of the soil, which decreases solubility of organic matter in the soil pore water. When sulfur deposition started to decline around 1990, DOC concentrations started rising, so DOC concentrations may be returning to pre-industrial levels (Monteith et al., 2007). A second possible cause of increasing DOC concentrations is altered land use. Worrall et al. (2012) studied DOC fluxes in the United Kingdom and showed that most DOC originated from organic soils (9.2 tons C km^-2^ y^-1^), but urban (6.7 tons C km^-2^ y^-1^), and grazed land (2.4 tons C km^-2^ y^-1^) can also contribute significantly to DOC in rivers. Regnier et al. (2013) estimate that on top of the 1.9 Pg C y^-1^ (see **Box 1**), inland waters receive another 0.8 Pg C y^-1^ from terrestrial soils because of anthropogenic perturbations, which mainly leads to higher amounts of CO_2_ emission, but also increased C storage and increased C transport to oceans. In a recent study by Noacco et al. (2017), a large data set (130 years) of DOC concentrations in the Thames basin was analyzed and it was concluded that 90% of the increase in DOC was linked to effects of increased urbanization, such as discharge of waste water, and land use changes like the conversion of grassland into farmland. However, changing land use can also decrease DOC concentrations. Around the Mississippi River, for instance, a significant part of wetlands, which could have released substantial amounts of DOC, have now been replaced with farmland. In the tributaries of the Mississippi River DOC subsequently decreased with 58%, leading to a lower downstream DOC concentration in the river (Duan et al., 2017). A third cause that could lead to increased DOC concentration is the effects of climate change such as: (i) increased precipitation (Wu et al., 2007; Brothers et al., 2014), but in other cases also (ii) decreased precipitation (Porcal et al., 2009) in combination with (iii) increased temperature (Fenner and Freeman, 2011), (iv) rising CO_2_ emissions, which can cause increased organic matter production by terrestrial (Zangerl and Bazzaz, 1984) and aquatic (Song et al., 2013) primary producers, and (v) increased nitrogen deposition (McElarney et al., 2010), although there are also studies that claim that DOC increases are caused by a decrease in nitrogen deposition (Musolff et al., 2017).

Altered land use and climate change can also change the quality of DOC. For example, DOC quality can change due to fragmentation of streams caused by drought. Vazquez et al. (2010) found that during fragmentation of a stream, the fluorescence index of DOC decreased, indicating that there was a higher contribution of autochthonous DOC. Moreover, they found that natural variation in DOC quality, like aromaticity, N content or biodegradability, at different locations in the stream became more pronounced after drought. Increased precipitation can also change DOC quality: in lakes there will be more terrestrial DOC as the climate becomes wetter, which reduces light and oxygen availability in the water (Kellerman et al., 2014). Altered land use can also affect DOC quality: Butman et al. (2014) concluded from a global data set of DOC in which the age was determined by carbon-14 dating that in highly populated areas, DOC had a higher age. Sources of this older DOC are probably C released due to land use changes, human waste water or fossil C products such as petroleum products. Concentrations of other anthropogenic compounds such as biocides, pharmaceutical products and remains of genetically modified crops are also increasing and affecting DOC composition in the water (Stanley et al., 2012). Urbanization can also affect DOC quality: it was found that in urban watersheds with high population density, the composition of DOC was different from natural or agricultural watersheds (Williams et al., 2016). The exact chemical differences were not studied, but DOC from urban watersheds appeared to be more humic-like, probably of microbial origin and more resistant to photo-degradation and may therefore be less likely to be broken down. In a study by Hosen et al. (2014), this was found as well, and they also found an increase in labile, protein like DOC and a decrease in natural humic-like DOC. They found, however that this urban DOC is more likely to be degraded, as microbial bioavailability of urban DOC is higher than bioavailability of natural DOC.

## The Effects of DOC on Macrophytes

### The Effect of Humic Substances on Light Availability to Macrophytes

**FIGURE 2:**
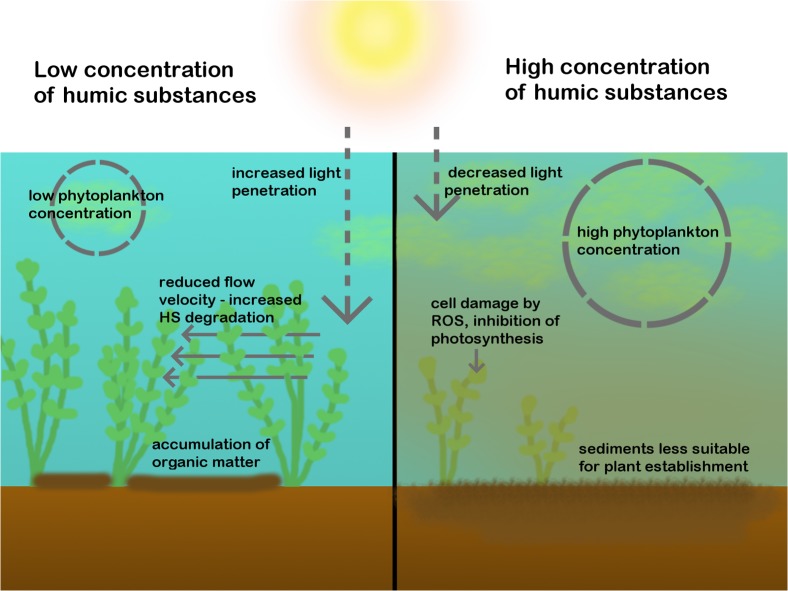
The effects of HS on macrophytes. On the left side, a scenario with low HS, high plant density and low phytoplankton density is shown. Plants receive enough light. Water flow is reduced, which causes OM accumulation within macrophyte patches and HS degradation. On the right side, a scenario with high HS, low plant density and high phytoplankton density is shown. Plants are damaged by HS, receive less light and HS makes the sediments less suitable for plant establishment.

Humic substances are the type of DOC that has the most pronounced effect on macrophytes and their effect has been studied most. HS are responsible for the brown color of water with a high DOC concentration (Evans et al., 2005). HS attenuate UV radiation and photosynthetically active radiation (PAR), and can thereby limit benthic primary production (Karlsson et al., 2009; Thrane et al., 2014), see **Figure 2**. DOC mainly attenuates the shorter wavelengths of PAR (the blue light) and the absorption coefficient decreases exponentially toward the longer wavelengths (Thrane et al., 2014). Although most studies about the effect of DOC on primary production focus on boreal lakes with limited macrophyte growth, there is evidence that macrophytes are affected as well by the effect of DOC on light quantity and quality; it can reduce their maximum colonization depth (Chambers and Prepas, 1988). In oligohumic soft water lakes (<4 mg L^-1^ DOC), macrophytes can grow at a depth of 12 m, whereas in meso- and polyhumic soft water lakes (4 to more than 40 mg L^-1^ DOC) this decreases to 1 m (Bociąg, 2003). This means that macrophytes are confined to the shallowest parts of the lake where additional disturbance from wave action may exclude some species (Szmeja and Bociąg, 2004). Not all species are equally vulnerable to changes in light quantity and quality. In Polish lakes, for instance, habitat characteristics of two *Ceratophyllum* species were studied, and it was found that water transparency and water color (mainly determined by HS) were important factors determining species occurrence. *Ceratophyllum demersum* was found in transparent waters, whereas *Ceratophyllum submersum* was found in more colored waters (>100 mg Pt L^-1^) (Nagengast and Gąbka, 2017). As a more general phenomenon, charophyte abundance decreased and bryophytes and vascular plants dominated during a wet period in a Polish lake in which conductivity decreased and DOC concentrations increased. DOC changed the color of the water and thereby reduced visibility. However, charophytes generally do not have higher light requirements than vascular plants. There are two alternative explanations for the decreased charophyte abundance: it has been suggested that the altered color of the water diminished the establishment of charophytes and provided an opportunity to competitors (Ejankowski and Lenard, 2015). Middelboe and Markager (1997) suggested that the negative effect on charophyte growth can also be caused by the fact that colored substances reduce the pH of the water. McElarney et al. (2010) found that DOC can reduce macrophyte abundance and diversity. In their study especially isoetids appeared to be sensitive to the change in water color, but they argued that DOC may have increased sedimentation of organic matter which increases sediment alkalinity and nutrient concentration, which is unfavorable to some macrophyte species. Besides light, other examples of indirect effects of DOC on macrophytes are discussed in Section “Indirect Effects of DOC on Macrophytes.” Effects on primary production in general and on macrophytes have been summarized in Supplementary Table S1. From this overview it can be concluded that most research on the effect of colored DOC on macrophytes focusses on lakes in northern Europe.

### Direct Effects of Humic Substances: Intracellular Damage

Although macrophytes are probably mainly indirectly affected by HS by light attenuation, HS may also directly affect macrophytes. HS form a wide class of substances with many different functional groups. This gives them the ability to interfere with nearly all biochemical processes that occur in freshwater organisms (Steinberg et al., 2008). Only a few studies have looked into these effects. There is evidence that small particles (<3.5 kDa) can be taken up by macrophyte cells (Steinberg et al., 2006), but it has not been studied yet in great detail. Inside cells they can, for example, lead to the formation of reactive oxygen species (ROS) that can damage the cells (see **Figure 2**). Production of oxidative stress enzymes by macrophyte cells significantly increased after exposure to DOC derived from decomposing beech leaves, which contain ROS (Grigutytë et al., 2009). Secondly, HS can interfere with photosynthesis (see **Figure 2**). This was demonstrated in *C. demersum* and is caused by quinoid structures in HS that take up electrons and thereby inhibit photosynthetic oxygen production. It has been hypothesized that macrophyte species may not be equally vulnerable to those quinoid structures (Pflugmacher et al., 2006), so species composition may be altered in very humic waters. Even though HS stress can cause damage to aquatic organisms, exposure to HS can also train the stress resistance of aquatic animals such as fish and nematodes. This improves their fitness in a fluctuating environment and can increase survivorship. Nematode *Caenorhabditis elegans* is even attracted to HS and actively seeks HS rich water (Steinberg et al., 2007). It is not known whether this intriguing phenomenon also applies to macrophytes, but Steinberg et al. (2008) suggest that mild HS stresses may be beneficial to specific plant organs caused by increased expression of anti-stress genes, resulting in multistress resistance. The chemical composition of DOC can vary substantially, based on catchment characteristics such as vegetation type (Amiotte-suchet et al., 2007), the presence of wetlands (Singh et al., 2015) and the type of soil (e.g., peat, mineral soil, anthropogenic influences) (Sachse et al., 2005). Steinberg et al. (2006) tested the response of different aquatic primary producers to DOC from different origins and found that the primary producers were not equally sensitive. They suggest that primary producers may adapt to the DOC type from their native environment.

### Defense Mechanisms Against HS

Macrophytes have developed defense mechanisms against HS and other environmental stresses. Studying the production of defensive compounds may help to find the molecular mechanisms behind the cellular damage caused by HS, interaction with other stressors and the way macrophytes cope with this. Omic technologies may be a valuable technique to gain more understanding in this field (Van Aggelen et al., 2010). Although most studies using omics focus on terrestrial plants, omics are increasingly applied to marine macrophytes (Kumar et al., 2016) and there are also a few examples of studying stress tolerance using omics in freshwater macrophytes.

A general stress response is the production of defense proteins like HSP70, a heat shock protein. This protein, or similar proteins, are present in virtually all living organisms and aim to protect cells from thermal or oxidative stress (El Golli-Bennour and Bacha, 2011). In macrophytes HSP70 expression in response to HS has not been tested, but in algae (Bierkens et al., 1998), fish and invertebrates (Steinberg et al., 2006) exposure to HSs leads to an increase of the concentration of HSP70. Other substances have been found to affect heat shock protein expression in macrophytes: Tukaj et al. (2011) found that HSP70 was induced in *Lemna minor* when it was exposed to different chemicals like heavy metals, polycyclic aromatic hydrocarbons and herbicides. This means that HSP70 may be used as biomonitor to see whether DOC causes stress in macrophytes, provided that other parameters such as temperature are kept constant. Another way macrophytes protect themselves from oxidative stress is the use of detoxification mechanisms like antioxidant enzymes and ROS-scavenging proteins (Chalanika De Silva and Asaeda, 2017). Acquired stress tolerance can be heritable to next generations, although heritability of HS tolerance has not yet been studied in macrophytes. In cladocerans, however, there is evidence of epigenetic inheritance: when they were exposed to HS, the percentage of methylated DNA increased. It has also been found that resistance to HS stress is transferred to the next generation, so it has been suggested that this may be caused by epigenetics (Menzel et al., 2011). Studying epigenetics in ecotoxicological research is relatively new but as it may explain inherited changes in the phenotype caused by environmental stress and stress adaptation it may be an interesting approach in aquatic ecology (Vandegehuchte and Janssen, 2014).

### Indirect Effects of DOC on Macrophytes

Although high concentrations of DOC are disadvantageous to macrophytes, DOC can also positively affect macrophytes by mitigating the effect of stressors. DOC attenuates UV-B radiation (Scully et al., 1995) that can decrease growth rates and damage DNA of charophytes (de Bakker et al., 2005). DOC may also stimulate heterotrophic bacteria by attenuating UV-B radiation and providing a food source, leading to faster DOC degradation, but on the other hand, UV-B radiation can transform recalcitrant DOC and make it more accessible to bacteria (Karentz et al., 1994). Another positive effect of DOC, or more specifically, polyphenols (major building blocks of HS) is that they can inhibit cyanobacteria and thereby can contribute to controlling blooms (Steinberg, 2014). DOC can also mitigate toxicity of anthropogenic pollutants like anthracene in macrophytes (Gensemer et al., 1999) and protect macrophytes against harmful heavy metals. Some chemical functional groups in humic acids, a subgroup of HS, have a negative charge, such as carboxylic and phenolic groups, which can bind to positively charged metal ions (Christl et al., 2001). Heavy metals such as copper, cadmium (Wang et al., 2010), lead (Kruatrachue et al., 2002), and zinc (Bunluesin et al., 2006) are taken up by macrophytes and can lower chlorophyll content. HS in the sediments bind to heavy metal and thereby significantly reduce accumulation of heavy metals in macrophytes (Wang et al., 2010). This appears to be beneficial to macrophytes, but others argue that especially binding of metals to allochthonous DOC, which has a higher binding capacity than autochthonous DOC due to the higher HS content, may be harmful to macrophytes. If there is a high concentration of allochtonous DOC, heavy metals may, instead of being adsorbed by the sediments, be bound to DOC and stay in the water column. When DOC is degraded, the heavy metals are released in the water column and this may be detrimental to aquatic organisms in general (Zhang et al., 2013). DOC can also negatively affect primary producers by enhancing mercury accumulation in macrophytes and epiphytes. Mercury can bind to sulfide and precipitate, but when it mercury binds to DOC, it will stay in solution. DOC may also stimulate mercury methylating bacteria. Methylated mercury can accumulate in the food chain; in most fish species more than 95% of the mercury is methylated (Ravichandran, 2004). Both methylated and unmethylated mercury can accumulate in macrophytes, and in epiphytes even higher concentrations can be found. Water level fluctuations and higher temperatures also stimulate mercury uptake, so under climate change mercury concentrations in macrophytes and epiphytes are expected to rise (Hamelin et al., 2015).

Dissolved organic carbon can also change soil properties, making the sediment more gelatinous and hydrated, which limits macrophyte establishment (Bociąg, 2003). DOC concentrations often correlate with CO_2_ concentrations in freshwater ecosystems (Sobek et al., 2003). DOC can be converted to CO_2_ by biodegradation (bacteria break down DOC) or by photodegradation (DOC is broken down by UV radiation). With increasing DOC concentrations, the fraction of biodegradable DOC appears to be constant, but the proportion of photodegradable DOC is enhanced as the input of terrestrial DOC increases, leading to more CO_2_ production (Lapierre et al., 2013). This can be beneficial to macrophytes as CO_2_ is often limiting (see paragraph 2), but high levels of CO_2_ in the water can lead to acidification and dominance of macrophyte species that only use CO_2_ as their inorganic C source. Species adapted to low CO_2_ concentrations such as isoetids lose their advantage and may disappear (Spierenburg et al., 2009). DOC can also bind to phosphorus (P) and iron (Fe), although it is not fully understood how binding of DOC to P and Fe affects their bioavailability, Findlay and Sinsabaugh (2003) suggest that reactivity of P and Fe is reduced if it is bound to DOC.

The concentration of DOC in water can also indirectly affect nitrogen availability to macrophytes. DOC serves as an energy source to denitrifying bacteria, so if there is a sufficient amount of nitrate in the water DOC can stimulate denitrification and therefore reduce nitrate availability (Taylor and Townsend, 2010). However, microbes that carry out dissimilatory nitrate reduction to ammonium (DNRA) are stimulated by high C/N ratios in the water, so this may favor conversion of nitrate to ammonium instead of denitrification (Tiedje, 1988). Ammonium is by most macrophyte species preferred over nitrate as source of nitrogen (Feijoó et al., 2002).

Altogether, it appears that DOC can have various indirect positive and negative effects on macrophyte growth. The net effect on macrophytes does not only depend on the concentration of DOC but also, for example, on the quality of DOC (HS content), intensity of UV radiation in the water and presence of microbes that degrade DOC. Moreover, the net effect of DOC on primary production largely depends on the characteristics of the aquatic system. For example, in boreal lakes with low productivity that are supersaturated with CO_2_, the net effect is negative: elevated CO_2_ concentrations due to DOC degradation do not lead to increased productivity because CO_2_ is not limiting and DOC diminishes light availability (Hessen et al., 2017).

## The Effects of Macrophytes on Aquatic Carbon

The relationship between carbon and macrophytes is not one-way; macrophytes increase sedimentation of organic C, they produce DOC, and take up inorganic C. Macrophytes contribute to sedimentation of carbon by taking C out of the water and sinking to the bottom after senescence (Flanagan et al., 2006). The physical structure of macrophytes also contributes to the removal of C from the water column: macrophytes reduce flow velocity and this causes accumulation of organic matter within macrophyte patches (Schoelynck et al., 2012). Still, carbon burial efficiency can also be reduced by the presence of macrophytes. Brothers et al. (2013) found that in an algae-dominated shallow lake, 80% of the amount of carbon entering the lake was buried in the sediments, whereas in macrophyte-dominated lakes this was only 40%. This can be explained by the fact that macrophytes provide bacteria in the sediments with oxygen which leads to enhanced C mineralisation. DOC release and inorganic carbon uptake by macrophytes are explained in next paragraphs.

### DOC Release by Macrophytes

When macrophytes grow, <1–10% of the amount of C they fix photosynthetically is released again as DOC (Carpenter and Lodge, 1986). Macrophytes can therefore be an important DOC source, yet most studies on autochthonous DOC only focus on algae (Findlay and Sinsabaugh, 2003). Søndergaard (1981) studied DOC release by several macrophyte species and concluded that it mostly consists of small (<1000 Daltons) and a smaller fraction of large (>10000 Daltons) molecules, depending on the plant species. Small molecules that are released can include amino acids and simple sugars, especially glucose. DOC release appears to be related to photosynthesis; in dark conditions DOC production is only 1% of DOC production in light conditions (Søndergaard, 1981). Moreover, in fast growing species, the rate of DOC release is higher than in slower growing species (Thomas and Kowalczyk, 1997). The effect of nutrient availability on DOC production is not clear. Takamura et al. (2003) found that *Trapa japonica* only causes DOC enhancement in the water when nutrient concentrations are high. However, Demarty (2009) did not find a correlation between DOC production by *Myriophyllum spicatum* and *Potamogeton* spp. and nutrient concentrations. Lastly, there is a relationship between the amount of inorganic C in the water and DOC release by the free floating macrophyte species *L. minor*. When there is a limited amount of inorganic C in the water, DOC release is higher than when there is an excess of inorganic C, even though macrophyte growth is impaired under low inorganic C conditions. It was suggested that the stress caused by the low inorganic C concentrations may have led to DOC leakage from the plants (Baker and Farr, 1987).

The contribution of macrophytes to the total amount of DOC in the water varies. Especially in lotic systems, the DOC contribution by macrophytes is small (1–20% of the total amount of DOC), probably because of DOC degradation by epiphytic bacteria and algae (Thomas, 1997), or insignificant (Hummel and Findlay, 2006). This may also be caused by the relatively low abundance of macrophytes in rivers compared to, e.g., wetlands. In wetlands (Briggs et al., 1993) and shallow lakes (Lapierre and Frenette, 2009) macrophytes can contribute significantly to DOC concentrations in water and organic C release by emergent macrophytes can even be in the same order of magnitude as organic C input from the catchment. In Lake Frisksjön in Sweden for example, organic C input from the catchment is 9600 kg C y^-1^ and production by emergent macrophytes is 6000 kg C y^-1^ (Sobek et al., 2006). Macrophyte DOC production is dependent on the season: in summer, when macrophyte biomass reaches its climax, macrophytes can cause large increases in DOC concentrations. At this time they can also alter the composition of DOC, as they primarily release carbohydrates whereas allochthonous DOC contains more humic and protein-like material (Catalán et al., 2014).

It is not exactly known why macrophytes release DOC, but there are a few hypotheses. The first hypothesis is the overflow mechanism, a passive mechanism which has been demonstrated in planktonic algae. The algae excrete sugars they produce during photosynthesis, when nutrient availability is limited (Jensen, 1984). It has also been hypothesized that macrophytes may actively release DOC. Some species excrete DOC from their roots to stimulate bacterial (Catalán et al., 2014) or endomycorrhizal (Wigand et al., 1998) growth and activity in the sediment in order to obtain more nutrients. It has also been suggested that DOC release serves as a C concentrating mechanism when CO_2_ is limiting, which works as follows: Demarty (2009) found that DOC release is positively linked to HCO_3_^-^ uptake, which is one of the strategies used by macrophytes to avoid inorganic C deficit (see section “CO_2_”). It has been suggested that the type of DOC released by the plant is carbonic anhydrase, an enzyme involved in HCO_3_^-^ use. However, DOC released by macrophytes mostly consists of small compounds, whereas carbonic anhydrase is a nitrogenous high weight compound and only 10% of the DOC falls into that category. Another form of DOC released by macrophytes is allelochemicals that serve to inhibit phytoplankton growth. This topic has been reviewed by Hilt and Gross (2008) and it can be concluded that there are at least 37 macrophyte species that produce allelochemicals, like *Myriophyllum, Ceratophyllum, Elodea, and Najas.* Most of the allelopathic compounds have not been identified, but at least part of them are polyphenols. It is hypothesized that those compounds are also involved in defense against herbivores and infections (Gutierrez and Mayora, 2015). Diatoms and cyanobacteria appear to be more sensitive to the allelochemicals than chlorophytes. Epiphytes are targeted as well, but it has been suggested that they have developed resistance against allelopathic substances from macrophytes (Hilt and Gross, 2008). Macrophytes can also diminish phytoplankton growth by limiting their nutrient availability (see section “The Effects of Macrophytes on DOC Concentrations”).

Dead macrophyte tissue also releases DOC. This can occur because of cell death, but also when cells are damaged by grazers or viral lyses (Findlay and Sinsabaugh, 2003). The nature of this DOC depends on the macrophyte species; it can differ, for example, in colour and C:nutrient ratio (Cuassolo et al., 2011), amount of humic-like matter and photoreactivity (Cuassolo et al., 2015) and percentage of proteins, amino acids and carbohydrates (Qu et al., 2013). Macrophyte DOC is less aromatic than allochthonous DOC but has a similar or higher aromaticity than phytoplankton DOC (Qu et al., 2013). In general, DOC released by macrophytes is relatively labile and rapidly decomposed by bacteria, compared to allochthonous DOC (Mann and Wetzel, 1996). However, They et al. (2012) found that a significant part of the DOC produced by macrophytes in a subtropical shallow lake remained in the water as unreactive molecules with a low molecular weight.

### The Effect of Macrophytes on DOC Concentrations

Freshwater macrophytes can also diminish DOC concentrations in several ways. Some species primarily take up nutrients from the sediments and this can reduce nutrient exchange between water and sediments. The resulting reduction in water column nutrient concentration leads to diminished growth of DOC producing organisms without roots such as phytoplankton, bacteria, and filamentous algae (Wigand et al., 2000). Moreover, macrophytes release oxygen from their roots, which stimulates bacterial decomposition of DOC (Mann and Wetzel, 2000). Macrophytes also increase the residence time of the water and this leads to a longer exposure to photo- and microbial degradation (see **Figure 2**). DOC forms an important food source for heterotrophic bacteria. Macrophytes serve as a substrate for those bacteria and epiphytic algae. The resulting communities of macrophytes and epiphytic bacteria and algae can be very productive and highly efficient with regard to DOC degradation (Wetzel and Sondergaard, 1998). Martin et al. (2005) found that the concentration of chromophoric DOC, the part of DOC that absorbs light in water, decreases as the water flows through macrophyte beds. A possible explanation may be the high abundance of epiphytic bacteria that degrade DOC. The interaction between heterotrophic bacteria and macrophyte-epiphytic algae complexes can also have implications for the aquatic food web. de Kluijver et al. (2015) found, by studying carbon isotope ratios (δ^13^C) in a Chinese lake, that carbon produced by macrophytes and epiphytic algae contributes to bacterioplankton (55%) and zooplankton (47%). Stimulation of zooplankton can, in turn, reduce abundance of phytoplankton and thereby maintain clear water.

## Interactions With Other Effects of Climate Change on C Cycling and Macrophytes

The effects of DOC on macrophytes are complex and depend mainly on the characteristics of the environment (see section “Defence Mechanisms Against HS”). In addition to that, climate change can also have other effects on the aquatic carbon cycle. For example, drought does not only affect DOC concentrations but also other dissolved compounds. This effect has been observed in Canadian lakes; decreased runoff rates lowered concentrations of iron, phosphorus, dissolved organic nitrogen (DON) and DOC in the water. This may have implications for the aquatic C cycle, more specifically the C sequestration in the sediments. One of the mechanisms of C sequestration is binding of DOC to amorphous iron. Since iron concentrations are even more reduced by drought than DOC concentrations, it has been suggested that in this way drought may lead to a decrease in C sequestration (Dillon and Mollot, 2005). Temperature can also affect carbon cycling. When temperatures are raised, community respiration increases; e.g., in an Alpine river, benthic community respiration increased by 20% when temperature was raised by 2.5°C (Acuña et al., 2008). When it is warmer, gross and net photosynthesis rates, as well as plant respiration are reduced, but heterotroph respiration rates increase. This means that more C stored in the system is now emitted as CO_2_ during warming (Moss, 2010).

Climate change and altered land use also can have profound effects on macrophytes. Especially changing temperatures can form a substantial threat to macrophytes (Short et al., 2016), but storms (wave action, mixing of water layers and nutrient loading), water level fluctuations (Zohary and Ostrovsky, 2011) increasing CO_2_ concentrations, increases in UV-B radiation, increasing salinity (Short et al., 2016) and eutrophication (Hossain et al., 2016) all affect macrophyte growth and the distribution of species. It can be concluded that mainly submerged macrophytes will decline, as they suffer most from the increases in water turbidity caused by increased DOC (Karlsson et al., 2009) and they may be outcompeted by phytoplankton and floating macrophytes that benefit from higher temperatures and from eutrophication, (Short et al., 2016). Different effects of climate change can have contrasting effects on macrophytes. For example, rising temperatures can enhance productivity of macrophytes in the littoral zone, whereas increased HS in the water decrease productivity (Rodríguez et al., 2015). In lakes, DOC may even act as a buffer against rising temperatures. As DOC attenuates light that heats up the water and enhances stratification of the water, so deeper parts of the lake are less exposed to higher temperatures (Read and Rose, 2013).

## Knowledge Gaps

Although many studies have looked into DOC in aquatic systems, there still are a number of research gaps, especially with regard to the link between DOC and macrophytes. Firstly, production of DOC by living macrophytes and the effect of elevated CO_2_ on DOC production are still poorly understood. It is still not clear why macrophytes produce DOC, what compounds it exists of and how much this process is affected by climate change. Secondly, there are research gaps with regard to the effects of DOC on macrophytes. Since the exact cause of the increase in DOC concentrations is not known, it is difficult to predict how DOC concentrations will develop in the future. Therefore, it is important that DOC concentrations are monitored over longer periods of time. Currently, most DOC research focusses on North America and Europe. Global monitoring campaigns are needed to provide more insight into the cause of DOC increases and role of freshwater ecosystems in the global carbon cycle.

Dissolved organic carbon covers a wide class of substances with many chemical functional groups. In most studies, those substances are not identified and it is not known whether and how they affect macrophytes on the cellular level. The quality of DOC also varies, depending on its source. DOC quality also appears to be different in densely populated areas (Williams et al., 2016). The chemical characteristics of this ‘anthropogenic DOC’ are not completely known and the number of studies looking at the changing quality of DOC due to anthropogenic disturbances is low. Gaining more knowledge about the nature of this changed quality of DOC and its effect on freshwater organisms is crucial to understanding the stability of freshwater ecosystems. In Section “Direct Effects of Humic Substances: Intracellular Damage” the effects of HS on macrophytes are discussed, however, anthropogenic DOC compounds such as pesticides, hormones and remains of genetically modified crops may pose a considerable threat to macrophytes although the exact consequences and scope of this problem are still poorly understood and require more research (Stanley et al., 2012).

It is also important to note that freshwater ecosystems are naturally heterogeneous systems. For example, rivers can be seen as a patchwork of different zones that vary in hydrogeomorphology and are affected by differences in the catchment and the climate. Those different patches may have different inputs of C and may vary in C processing rates (Thorp et al., 2006). In addition, drought may further increase those differences by decreasing connectivity between different parts of the river (Vazquez et al., 2010). This needs to be taken into account when studying the effects of changed DOC quality and quantity on macrophytes.

There is also a lack of knowledge about the fate of allochthonous DOC from different origins; whether it is degraded or not, how it is degraded and to what extent abiotic factors like light and nutrients play a role (Evans and Thomas, 2016). It was assumed that terrestrial, colored DOC is relatively resistant to degradation by microbes. However, laboratory experiments have indicated that when organic C is added to stream water, it is rapidly broken down to CO_2_ after it had entered the water during a storm (Goulsbra et al., 2016). It appears that this terrestrial, colored DOC is degraded by microbes, but the carbon use efficiency is low, meaning that the main part is converted to CO_2_ instead of microbial biomass (Fasching et al., 2014). Molecular characteristics are an important factor determining degradability of DOC. Oxidized, aromatic molecules are better degradable than reduced, aliphatic and N-containing molecules (Kellerman et al., 2015). Furthermore, DOC with a large molecular size and DOC originating from terrestrial plants appears to be more easily degraded than DOC from agriculture or wastewater (Bodmer et al., 2016). More knowledge on the degradability and residence times of DOC, and therefore also the degree of exposure to macrophytes can help to predict the effects on macrophytes. If DOC is degraded, this is not always beneficial to macrophytes. For example, photo-oxidation of DOC can lead to release of toxic trace metals that can be taken up by macrophytes (Porcal et al., 2009). Understanding the fate of DOC is also vital to understand the aquatic C cycle. If the quantity and quality of DOC are changed by climate change, this may have large effects on the extent of C sequestration in aquatic sediments and on aquatic CO_2_ emissions. Although it is important to study the fate of DOC, DOC itself can also be regarded as C sink for anthropogenically emitted CO_2_ as DOC production in algae increases under elevated CO_2_ concentrations (Song et al., 2013).

To conclude, in order to gain improved understanding of the effects increased quantity and quality of DOC has on macrophytes and to be able to conserve stable macrophyte populations, the following points have priority: (1) detailed modeling covering a large spatial scale can contribute substantially to understanding the effects of increasing DOC and changing climate on the aquatic C cycle (Porcal et al., 2009). (2) More inclusion of DOC quality and quantity in river management, especially in relation to the potential for riparian zones to buffer DOC rises (Stanley et al., 2012). As most studies on DOC focus on lakes in northern Europe (see Supplementary Table S1), it is also important to study DOC in other parts of the world and to include lotic systems. (3) Carrying out experimental studies to help predicting the morphological and physiological responses to changing DOC quantity and quality.

## Author Contributions

RR was the principal investigator of the study, was involved in all parts of the literature search, and wrote the manuscript. JS and PM were promotors of the study, financed and facilitated the work, wrote significant parts of the text, and proofread the entire manuscript.

## Conflict of Interest Statement

The authors declare that the research was conducted in the absence of any commercial or financial relationships that could be construed as a potential conflict of interest.
