# Supplementary material for: The Future of Freshwater Macrophytes in a Changing World: Dissolved Organic Carbon Quantity and Quality and Its Interactions With Macrophytes
Source: Front Plant Sci. 2018 May 14;9:629. doi: 10.3389/fpls.2018.00629 (PMC5960680; doi:10.3389/fpls.2018.00629)
Supplement: Supplementary file 1 [file Table_1.DOCX]

**Table S1:** An overview of the effects of coloured DOC on primary production and freshwater macrophytes, with information on the study locations, their climate and chemistry.

| DOC concentration | DOC type | Macrophyte species | Effect on macrohpytes | Location | Geology and climate | Type of system | Reference |
| --- | --- | --- | --- | --- | --- | --- | --- |
| *Effect of light limitation on primary production in general* | | | | | | | |
| 2.4-16.8 mg l^-1^ | Not specified | General effect on primary production was measured | Light attenuation and mean depth explained 73% of benthic PP | Northern Sweden | Boreal climate | Oligo- or mesotrophic lakes | Karlsson et al., 2009 |
| 0.25-27.5 mg l^-1^ | Not specified | General effect on primary production was measured | A doubling in CDOM can decrease PP/PAR by 32% | Norway and Sweden | Boreal climate | Oligo- or mesotrophic lakes. pH >5 | Thrane et al., 2014 |
| *Effect of light limitation on macrophytes* | | | | | | | |
| Between <4.0 and >40.0 mg l^-1^ | Humic substances | Bryophyta, cormophyta, characeae | Macrophytes grow at 12 meter depth in oligohumic lakes and at 1 meter depth in humic lakes | Northwestern Poland | Temperate climate, landscape of moraines | Oligo- or mesotrophic soft water lakes | Bociag, 2003 |
| Between <4.0 and >40.0 mg l^-1^ | Humic substances | *Isoetes lacustris, Lobelia dortmanna, Sphagnum denticulatum, Fontinalis antipyretica* | Settlement and aggregation density indexes are lower in humic lakes, especially in deep and central parts | Northwestern Poland | Temperate climate, landscape of moraines | Oligo- or mesotrophic soft water lakes | Szmeja & Bociag, 2004 |
| Secchi disk transparency: 0.14-28.2 m  Water colour was measured as well (results not mentioned) | Humic substances | Bryophytes, charophytes, caulescent angiosperms, rosette-type angiosperms, *Isoetes* spp. | Negative correlation between water colour light intensity, some species are more vulnerable than others | Denmark, Finland, Norway, Scotland, Canada, New Zealand, The Netherlands, Poland, US | Various | Danish lakes: Shallow and often eutrophic. Non-danish lakes differed in nutrient status | Middelboe & Markager |
| 0.36-22.47 mg l^-1^ | Humic substances | *Ceratophyllum demersum* and *C. submersum* | *C. demersum* was found in relatively transparent water, *C. submersum* in more coloured water | Western Poland | Temperate climate | Oxbow lakes, post-exploitation ponds, periodical overflow areas and small lakes | Nagengast & Gabka, 2017 |
| Water colour: 0.2-65.4 mg Pt l^-1^ | Humic substances | Bryophytes, charophytes and vascular plants | Charophyte establishment decreased when DOC concentrations were higher, which provided an opportunity to bryophytes and vascular plants | Eastern Poland | Temperate climate | Mesotrophic, hard water lake | Ejankowski & Lenard, 2015 |
| Mean annual concentration: 14.81 mg l^-1^ ± 0.84 SE | Humic substances | Bryophyta, characeae, elodeids, isoetids, macroalgae, potamogetonaceae, callitrichaceae, cyperaceae, lemnaceae, nymphaeaceae, sparganiaceae | Increases in DOC have a negative impact on macrophyte abundance and diversity because of light limitation and effects on lake chemistry | Northern Ireland | Temperate oceanic climate | Softwater, humic lakes | McElarney et al., 2010 |
